# Impact of Smoking on the Healing of Apical Periodontitis after Nonsurgical Endodontic Treatment

**DOI:** 10.1055/s-0043-1761451

**Published:** 2023-03-28

**Authors:** Ema Paljevic, Ivana Brekalo Prso, Jelena Vidas Hrstic, Sonja Pezelj-Ribaric, Romana Persic Bukmir

**Affiliations:** 1 Department of Endodontics and Restorative Dentistry, Faculty of Dental Medicine, University of Rijeka, Rijeka, Croatia; 2 Dental Medicine and Health Osijek, Josip Juraj Strossmayer University of Osijek, Osijek, Croatia; 3 Department of Oral Medicine, Faculty of Dental Medicine, University of Rijeka, Rijeka, Croatia

**Keywords:** cigarette smoking, periapical periodontitis, root canal therapy, treatment outcome

## Abstract

**Objectives** The aim of this prospective study was to compare the healing of periapical bone between smokers and nonsmokers after root canal therapy. The effects of duration and intensity of smoking on the healing rate of apical periodontitis were analyzed.

**Materials and Methods** Fifty-five smokers were included in this study. The control group consisted of healthy nonsmokers who matched the smoker group in age and sex. Only teeth with a favorable periodontal prognosis and adequate coronal restoration were included in the study. The periapical status of treated teeth was assessed using the periapical index system at follow-ups after 6 and 12 months.

**Statistical Analysis** The chi-squared test and Mann–Whitney U test were used to assess the changes in periapical index score at baseline and in subsequent time intervals between the two groups examining dichotomized and ordinal data, respectively. Multivariate logistic regression analysis was used to test the association of independent variables age, gender, tooth type, arch type, and smoking index with the outcome variable. The outcome variable was set as the presence versus absence of apical periodontitis.

**Results** The analysis at 12-month follow-up revealed a significantly higher healing rate in control group than in smokers (90.9 vs. 58.2; χ2 = 13.846; *p* < 0.001). Smokers had significantly higher periapical index scores than the control group ( *p* = 0.024). The multivariate logistic regression analysis demonstrated that an increase in the value of the smoking index significantly increases the risk of apical periodontitis persistence (odds ratio [OR] =7.66; 95% confidence interval [CI]: 2.51–23.28; *p* < 0.001) for smoking index < 400 and (OR = 9.65; 95% CI: 1.45–64.14; *p* = 0.019) for smoking index 400 to 799.

**Conclusion** The results from this study show a lower rate of apical periodontitis healing in a group of smokers at 1-year follow-up. Delayed periapical healing seems to be associated with the cigarette smoking exposure.

## Introduction

Apical periodontitis (AP) is an acute or chronic inflammation of the apical periodontium caused by bacterial infection of the root canal system. 1 Diagnosis is based primarily on radiographic findings of periradicular radiolucency, sometimes accompanied by clinical signs. 2 AP often presents as a chronic, asymptomatic condition leading to underestimation of its prevalence and burden.

Healing of periapical bone lesions is a lengthy process, monitored clinically and radiographically, and influenced by local and systemic predisposing factors. 3 Tobacco smoking is recognized as a global public health problem that negatively affects both systemic and oral health. 4 5 Smoking habit has been suggested as a modulating factor that could negatively affect the healing of periapical bone lesions through multiple mechanisms. 6 It affects the microvasculature by decreasing nutrient and oxygen levels, 7 limiting pulp defense mechanisms, and contributing to its necrosis. 8 Smoking impedes tissue repair leading to fibroblast dysfunction and impaired collagen synthesis. 9 It can alter the immune response to infections by suppressing immune cell functions and causing a stronger systemic inflammatory response. 10

According to the 2015 study by the Croatian Institute for Public Health, cigarette smoking is a widespread habit in Croatia. The questionnaire revealed that 31.1% of the Croatian population consumes cigarettes (35.3% smokers among men and 27.1% among women), 27.5% of them daily. 11 Similarly, smoking is a common habit globally with estimates of 32.6% adult male smokers and 6.5% female smokers in 2020. 12

Previous studies investigating the effects of smoking on endodontic variables were cross-sectional studies with contradictory conclusions. 13 14 15 These studies did not consider the presence of confounding variables such as periodontal disease affecting the tooth, diabetes mellitus that could affect the healing process or the socioeconomic status (SES) of the patient. Healing of AP can take up to 4 years, which prevents the evaluation of treatment outcome through a cross-sectional study design, as it is unclear whether the lesion is persistent or in a healing phase. 16

The aim of this cohort study was to compare the healing of periapical bone after nonsurgical endodontic treatment between smokers and nonsmokers and to assess the influence of smoking intensity and duration on the healing rate of AP at 1 year follow-up. We hypothesized that smoking habit is associated with prolonged or absent healing of AP.

## Materials and Methods

This prospective study was conducted at the Department of Endodontics and Restorative dentistry University Dental Clinic, Rijeka Clinical Hospital Centre, Rijeka, Croatia. Adult patients who agreed to participate by signing an informed consent form were enrolled in the study. The study was conducted in accordance with the Declaration of Helsinki and approved by the Ethics Committee of the Rijeka Clinical Hospital Centre (003-05/20-1/131). This study is registered in ClinicalTrials.gov registry with an associated identifier number NCT04812171.

### Participants

**Fig. 1:**
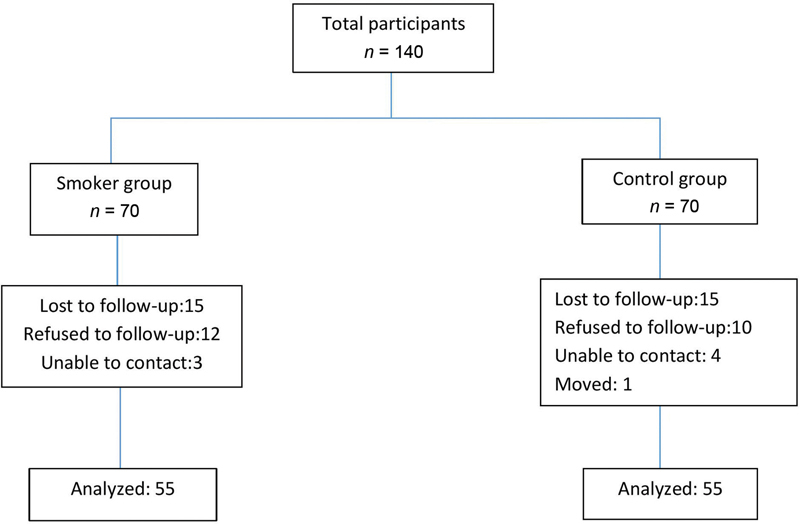
The flowchart for patient recruitment and treatment.

Fig. 1 shows the flowchart of participants through stages of recruitment and treatment. Only participants with a radiologically confirmed diagnosis of AP were included in the study, and strict inclusion and exclusion criteria were applied. Each participant provided one tooth into the research that had not been previously treated. Data on the health status of each participant were collected using an FDI Health Questionnaire, and data on smoking habits and SES were collected using a self-administered structured questionnaire. Participants were classified as smokers if they answered in the affirmative to the questions: “Have you consumed at least 100 cigarettes in your lifetime,” and “Do you currently smoke?” The smoking index is a unit for measuring cigarette consumption over a long period and was calculated using data on duration of smoking habit in years and cigarette consumption per day (CPD). 17 It quantifies smoking exposure and consists of the following categories: nonsmoker, less than 400, 400 to 799, and 800 and over. 18 Smoking intensity was assessed using data on CPD, classifying heavy smoking as 20 or more CPD and mild smoking as less than 20 CPD. 19 A cutoff was set at 20 cigarettes because differences in CO _2_ , cotinine, and nicotine levels were observed between a group of smokers who smoked 20 or less CPD and a group who consumed more CPD. 20 Participants diagnosed with a systemic disease or taking medications known to alter immunologic response or bone metabolism were excluded from the study. Also, former and occasional smokers, pregnant patients, and individuals who refused to participate were excluded from the study. The control group consisted of healthy nonsmokers who matched the smoker group in age and gender. Even though age and gender were not identified as confounding factors, previous studies observed that the prevalence of AP increases with age and that men has a higher percentage of teeth with AP. 14 21 Regarding the SES, participants provided information on their education level, monthly household income, self-assessed SES, and urbanization level.

To minimize the role of confounding factors, only teeth with a favorable periodontal prognosis (< 5 mm attachment loss and marginal bone loss of < one-third of the root length) were included in the study. 22 23

### Methods

AP was diagnosed based on clinical and radiographic examination. Endodontic specialists performed root canal treatments according to the standardized endodontic treatment protocol, which includes administration of local anesthesia and isolation with rubber dam. The teeth included in the study had well-performed root canal treatment with homogeneously obturated canals up to 1 mm from the radiographic apex and direct or indirect coronal restoration with clinically and radiographically well-sealed margins.

Analysis of marginal bone loss was performed using periapical radiographs at baseline and both follow-ups. Marginal bone loss was classified as more or less than one third of the root length. 23

Follow-up examinations were arranged 6 and 12 months after root canal treatment. Teeth were evaluated clinically by percussion and palpation tests, periodontal probing and examination of the coronal restoration. Immediately after treatment and at each follow-up visit, a standardized periapical radiograph of each tooth was taken using a sensor holder and the Planmeca ProX intraoral X-ray unit and Planmeca Romexis software (Planmeca Oy, Asentajankatu 6, Helsinki, Finland).

The periapical status of treated teeth was assessed using the periapical index system (PAI). 24 A PAI score was determined by one calibrated examiner using visual references for each of the five categories on an ordinal scale. The highest PAI value of all roots was used to determine the periapical status in multirooted teeth. Kappa values for inter-and intra-examiner agreement were 0.75 and 0.81 respectively. The PAI scores were dichotomized, recording AP as absent (PAI scores 1 and 2) and present (PAI scores 3, 4, and 5). Clinically, AP was assessed as healed in asymptomatic teeth, not sensitive to palpation or percussion.

### Sample Size Determination

Using Medcalc software size determination was used based on input of previously reported prevalence of AP in smokers and nonsmokers. 25 The computation was performed at *p* -value less than 0.05 and power 0.10 and resulted with a minimum sample size of 51 participants in each group. Keeping in mind the patient dropout rate, 70 patients per group were recruited.

Statistical analysis was carried out using IBM SPSS 26 (IBM Corp, Armonk, New York, United States) and MedCalc statistical software (MedCalc Software Ltd., Ostend, Belgium) with level of statistical significance set at *p* -value less than 0.05. The Kolmogorov–Smirnov test was used to test data for normal distribution. Because the data were not normally distributed, nonparametric tests were applied.

The chi-squared test and Mann–Whitney U test were used to assess the changes in PAI score at baseline and in subsequent time intervals between the two groups examining dichotomized and ordinal data, respectively. Multivariate logistic regression analysis (enter model) was used to test the association of independent variables age, gender, tooth type, arch type, and smoking index with outcome variable.

## Results

A hundred and ten patients were included in the study, 78 women and 32 men (70.9 vs. 29.1%) ranging in age from 18 to 66 years (median: 35.0; interquartile range: 29–46). The basic group characteristics are presented in Table 1 . There were no significant differences between the two groups regarding age, gender, tooth type, SES or PAI scores at the baseline.

**Table 1:** Basic group characteristics

Variables	Smokers ( *n* = 55)	Control ( *n* = 55)	Statistical test	Statistics
**Age** (median, interquartile range)	34 (28.25-45.5)	35 (29.0-45.75)	Mann–Whitney U test	0.900
**Gender**				
Male	16	16	Chi-squared test	χ2 = 0.044 *p* = 0.834
Female	39	39		
**Tooth type**				
Single rooted	8	8	Chi-squared test	χ2 = 2.286 *p* = 0.319
Premolars	13	7		
Molars	34	40		
**Arch type**				
Maxilla	22	18	Chi-squared test	χ2 = 0.354 *p* = 0.552
Mandible	33	37		
**Level of education**				
Elementary school High school University	2 37 16	0 35 19	Chi-squared test	χ2 = 2.304 *p* = 0.316
**Average monthly household income expressed in Kunas**				
1,000–4,000 4,000–6,000 6,000–10,000 Above 10,000	6 14 14 19	5 12 20 16	Chi-squared test	χ2 = 1.561 *p* = 0.668
**Self-assessed SES**				
Below average Average Above average	6 26 22	3 33 18	Chi-squared test	χ2 = 2.231 *p* = 0.328
**Urbanization level**				
Urban area Suburban area Rural area	30 17 8	29 17 8	Chi-squared test	χ2 = 0.08 *p* = 0.996
**PAI scores baseline (mean rank)**	53.19	57.81	Mann–Whitney U test	*p* = 0.415

Abbreviations: PAI, periapical index; SES, socioeconomic status.

On average, smokers consumed 12.22 cigarettes per day (median 12.0; interquartile range: 5–20) and most of them (72.7%) were categorized as “mild smokers.” Duration of a smoking habit ranged from 1 to 40 years (median: 15.0; interquartile range: 8–22).

Men consumed significantly more cigarettes per day in comparison to women ( *p* < 0.001). Significantly more men were categorized as “heavy smokers” in comparison to women (50 vs. 17.9%, *p* = 0.015) consuming 20 or more cigarettes per day. However, no significant difference in healing outcome was found with regard of the gender of the smokers ( *p* = 0.43).

The chi-squared test was used to analyze the difference in healing rate in smokers and control group at 6-month and 12-month follow-up ( Table 2 ). There was no significant difference in healing rate at 6-month follow-up. Conversely, analysis at 12-month follow-up revealed significantly higher healing rate in control group than in smokers (90.9 vs. 58.2; χ2 = 13.846; *p* < 0.001). Analysis according to full-scale PAI also revealed difference only at this point. Smokers had significantly higher PAI scores than control group ( *p* = 0.024; Table 3 ).

**Table 2:** Treatment outcome in smoker and control group at the 6- and 12-month follow-up

Group	Healed (6-month follow-up) *n* (%)	Not-healed (6-month follow-up) *n* (%)	Chi-squared test	Healed (12-month follow-up) *n* (%)	Not-healed (12-month follow-up) *n* (%)	Chi-squared test
Smokers	20 (36.4)	35 (63.6%)	χ2 = 0.341 *p* = 0.559	32 (58.2%)	23 (41.8%)	χ2 = 13.846 *p* < 0.001
Control	24 (43.6)	31 (56.4%)		50 (90.9%)	5 (9.1%)	
Total	44 (40.0)	66 (60.0%)		82 (74.5%)	28 (25.5%)	

**Table 3:** Difference between smoker and control group in PAI at the 6- and 12-month follow-up

Group	PAI (6-month follow-up)	Mann–Whitney U test	PAI (12-month follow-up)	Mann–Whitney U test
Smokers	3 (2–3)	*p* = 0.295	2 (1–3)	*p* = 0.024
Nonsmokers	3 (1–3)		1 (1–2)	

Abbreviation: PAI, periapical index.

Multivariate logistic regression analysis was used to test the association age, gender, tooth type, arch type, and smoking index with outcome variable. The dichotomous outcome variable was set as the AP healing versus AP nonhealing at 12-month follow-up ( Table 4 ). The only variable significantly associated with the outcome variable was the smoking index. The regression analysis demonstrated that the risk of AP persistence significantly increases with increase in the value of the smoking index (odds ratio [OR] =7.66; 95% confidence interval [CI]: 2.51–23.28; *p* < 0.001) for smoking index less than 400 and (OR = 9.65; 95% CI: 1.45–64.14; *p* = 0.019) for smoking index 400 to 799.

**Table 4:** Multivariate logistic regression analysis of independent variables on the AP healing outcome

Variable	Total	AP healed, *n* (%)	AP not healed, *n* (%)	OR	95% CI	*p* -Value
**Age**	Continuous variable			0.98	0.94–1.03	0.389
**Gender**						
Male	32	23 (71.9)	9 (28.1)	1	Reference	
Female	78	59 (75.6)	19 (24.4)	0.84	0.30–2.39	0.748
**Tooth type**						
Anterior	16	13 (81.2)	3 (18.8)	1	Reference	
Premolar	20	15 (75.0)	5 (25.0)	1.04	0.18–6.07	0.964
Molar	74	54 (73.0)	20 (27.0)	1.66	0.35–7.86	0.525
**Arch type**						
Maxilla	40	30 (75.0)	10 (25.0)	1	Reference	
Mandible	70	52 (74.3)	18 (25.7)	1.09	0.39–3.02	0.873
**Smoking index**						
Nonsmoker	55	50 (90.9)	5 (9.1)	1	Reference	
< 400	47	27 (57.4)	20 (42.6)	7.66	2.51–23.28	<0.001
400–799	8	5 (62.5)	3 (37.5)	9.65	1.45–64.14	0.019

Abbreviations: AP, apical periodontitis; CI, confidence interval; OR, odds ratio.

## Discussion

To our knowledge, there are no previous prospective studies on the relationship between smoking and AP healing that would allow comparison with the results of the present study. However, the results are consistent with the findings of several cross-sectional studies that found a higher prevalence of AP in smokers. 14 25 26 27 The present study showed a significant difference in healing rate between smokers and nonsmokers (90.9 and 58.2%, respectively). A negative effect of smoking was also observed in a study investigating the relationships between smoking habits and periodontitis healing after mechanical periodontal therapy. 28 A lower AP healing rate in smokers could be attributed to the deleterious effect of cigarette consumption on the microvasculature, decreased pulp and periradicular tissue defense, and impaired tissue repair. 7 8 9

Several previous studies have examined the association between smoking intensity and tooth loss. A study conducted among middle-aged Finnish adults found an exposure-related association between smoking intensity and tooth loss. 29 This study was based on a cohort project and measured smoking exposure in pack-years, calculated by multiplying the number of packs of cigarettes smoked per day by the number of years the person has smoked, without specifying the cause of tooth loss. When the reasons for tooth loss were considered, intensity and duration of smoking habits were significantly associated with tooth loss due to periodontal disease. 30 The number of cigarettes consumed and duration of smoking were positively associated with tooth loss in a study conducted in Denmark. 31 Current smokers who consumed more than 15 cigarettes per day for more than 27 years had increased scores of missing teeth and associated OR compared with never smokers. In the present study, the smoking index was calculated using data on the number of cigarettes consumed per day and years of tobacco use. Higher values of the smoking index were associated with a 9.65-fold increase in the risk for the presence of AP compared to nonsmokers (95% CI: 1.45–64.14; *p* = 0.019). Since the persistence of AP ultimately leads to tooth loss, the results of this study are consistent with several previous studies that have identified smoking intensity and duration as a risk factor for tooth loss. 31 32 33

The overall healing rate at 6-month follow-up was 40%, while after 1 year almost 75% of the teeth examined were free of radiographic and clinical signs consistent with AP. A limitation of this study is the relatively short follow-up period of 12 month, considering that the European Society of Endodontology recommends that the lesion be assessed over a 4-year period. 16 A study by Huumonen and Ørstavik reported statistically significant healing up to 2 years after nonsurgical endodontic treatment, and although recall rates were low at 3 and 4 years, the trend of healing was confirmed. 34

Socioeconomic factors are associated with systemic and oral health and assert their influence through health-related variables. Individuals with lower SES reported a higher risk of tooth loss. 35 To avoid the confounding effect of socioeconomic variables, both groups were tested, and no significant difference was found with respect to SES.

Because the healing outcome was not influenced by age or gender, the results of this study are consistent with other studies observing variables that influence the outcome of nonsurgical endodontic treatment. 36 37

Chronic endodontic and periodontal inflammation share several common features and to exclude the influence of marginal periodontitis on the healing of AP, only teeth with a favorable periodontal prognosis were included in the study. Previous studies have examined the relationship between apical and marginal periodontitis and the effect of smoking on marginal bone levels. A significant difference in marginal bone level between smokers and nonsmokers was observed, with smokers having a more reduced marginal bone level. 38 39

In the present study, more female participants were found to seek endodontic treatment at secondary dental care. This could be a confounding factor since the representation of men (29.1%) and women (70.9%) was not even. This difference could be due to the health awareness of female participants who are more likely to seek dental care and attend check-ups. 40

## Conclusions

To the best of the authors' knowledge, this is the first prospective study to investigate the association between smoking habit and the healing of AP with strict inclusion and exclusion criteria. The results of this study show a significant association between smoking habit and prolonged healing of AP. Moreover, the odds of AP persistence increased with an increase in smoking exposure. The results of this study suggest that cigarette smoking may be a modulating factor that delays or inhibits periapical healing and influences clinical decisions and guidelines concerning smokers.
